# Pain: its prevalence and correlates among the oldest old

**DOI:** 10.1007/s40520-023-02653-y

**Published:** 2024-01-20

**Authors:** Josephine Bokermann, Hans-Helmut König, André Hajek

**Affiliations:** Department of Health Economics and Health Services Research, University Medical Center Hamburg-Eppendorf, Hamburg Center for Health Economics, Hamburg, Germany

**Keywords:** Pain, Aged, 80 and over, Oldest old, Prevalence, Correlates

## Abstract

**Background:**

There is very limited knowledge regarding pain among the oldest old.

**Aims:**

To investigate the prevalence and correlates of pain among the oldest old.

**Methods:**

Data were taken from the “Survey on quality of life and subjective well-being of the very old in North Rhine-Westphalia (NRW80+)”, including individuals living in North Rhine-Westphalia aged 80 years and over. Pain was categorized as no pain, moderate pain and severe pain. Its prevalence was stratified by sex, age groups, marital status, place of residence and education. A multinomial logistic regression analysis was conducted.

**Results:**

28.50% of the participants reported no pain, 45.06% moderate pain and 26.44% severe pain. Regressions showed that being 85 years or older and a better self-rated health status decreased the likelihood of moderate pain. Being 85–89 years old, being male, highly educated and a better self-rated health status decreased the likelihood of severe pain. The likelihood of moderate and severe pain increased with a higher number of chronic diseases.

**Discussion:**

Study findings showed a high prevalence of pain in the oldest old living in North Rhine-Westphalia, Germany. The likelihood of having moderate or severe pain was reduced among those who were older and presented with a better self-rated health but increased with a growing number of comorbidities. Severe pain was less likely among men and those with a higher education.

**Conclusion:**

This cross-sectional representative study adds first evidence of prevalence and correlations of pain among the oldest old. Longitudinal studies are required to further explore the determinants of pain in this age group.

## Introduction

The population worldwide and in Germany is getting older. For Germany, an increase of individuals aged 80 years or older from 5.4 million in 2018 to between 8.9 and 10.5 million in 2050 is expected [1]. Despite the ongoing increase of individuals aged 80 and over, there is restricted knowledge regarding pain in this age bracket. This especially applies to the perception, experience and correlates of pain [2–8].

Pain can be divided into two types: acute and chronic pain. While acute pain is defined as “an unpleasant sensory and emotional experience associated with, or resembling that associated with, actual or potential tissue damage” [9], chronic pain “lasts or recurs for longer than 3 months” [10]. The latter is likewise classified into two groups: primary chronic pain is induced by an illness itself and secondary chronic pain refers to a relationship with another preceding disease [10].

Various studies investigated the determinants of pain in different populations showing that age, female sex, low socioeconomic status and poor education are significant risk factors for developing and experiencing pain [11, 12]. Comorbidity, cognitive impairment and depression are likewise associated with pain [12–15]. Furthermore, physical activity leads to decreased pain [16–18]. Most of the existing studies did not focus on the oldest old but rather on individuals aged 65 or older. Although they partly included the oldest old, their analysis did not distinguish between them and younger participants [7, 19, 20].

Studies investigating individuals aged 80 years or older found a prevalence of pain ranging from 50.4% to 63% [20, 21]. The intensity of pain differs: Mallon et al. [20] showed that 26.4% of the oldest old had mild pain and 36.6% had moderate to severe pain while Landi et al. [21] found that 39.2% of the oldest old had mild pain and 11.2% had moderate to severe pain in their sample. Lower pain scores in the oldest old are associated with being male, being older, a lower number of coexisting conditions and less use of medications [20–22].

Due to the limited knowledge, the objective of the present study was to investigate the prevalence and correlates of pain among the oldest old in Germany. This knowledge is important because pain in turn can contribute to future disability, decreased mobility, institutionalization and mortality [23, 24]. Moreover, suffering from pain has a substantial impact on an individual’s quality of life and successful aging [25].

## Methods

### Sample

For the present study, data were taken from the “Survey on quality of life and subjective well-being of the very old in North Rhine-Westphalia (NRW80+)” which only included individuals of 80 years or older who had their main residence in North Rhine-Westphalia (NRW) which is the most populous state in Germany. Data were collected from August 2017 to February 2018 via computer-assisted personal interviews (CAPI) by trained interviewers and lasted around 90 min. In cases where individuals were willing to participate but not able to, proxy interviews were conducted. The NRW80+ study aimed to gain insights into the living environment of the oldest old, their socioeconomic status and health-related factors [26].

Through a multistage sampling procedure, a representative sample was drawn. This led to the inclusion of 94 communities in NRW. Each registration office of the selected sampling points provided 400 randomly drawn addresses of the target population. Following a disproportional sampling strategy, 8040 individuals living in private homes and institutionalized settings were contacted. The response rate was 23.4% which yielded a total sample of 1863 individuals of which 1702 interviews were carried out with the target person itself and 176 interviews with a proxy. Considering the age of the target group, place and duration of the interviews as well as no usage of incentives for participation the response rate is regarded as satisfactory [27].

The NRW80+ study was approved by the ethics committee of the Medical Faculty of the University of Cologne (No. 17–169) and carried out in accordance with the Helsinki Declaration of 1975 (in its most recently amended version). Written informed consent was obtained from all participants included in the study.

### Dependent variable

The primary outcome variable in this study was pain which was measured with a single-item question taken from the SF-8 questionnaire: “If you had pain during the last 4 weeks, how severe was your pain?”. Possible answers on a 5-point Likert scale were: “no pain”, “mild”, “moderate”, “severe” or “very severe”. It was also acceptable to respond with “I don’t know.” which was done by 12 participants.

Following previous research based on large cohort studies (SHARE; [6, 20, 28]), we divided pain into three categories: no pain, moderate pain (combining mild and moderate pain) and severe pain (combining severe and very severe pain).

### Independent variables

The following independent variables were included in the analysis: age, marital status, education, place of residence, self-rated health status and number of chronic diseases. Age was retrieved through register data and summarized into age groups from 80–84 years, 85–89 years and ≥ 90 years to be able to determine differences of prevalence between them for clinical relevance. Concerning marital status, the following answers were possible: married, married but separated, divorced, widowed and single. For this study, a binary variable was created to differentiate between those who are married (and living together) and all other groups. The level of education was quantified using the “International Standard Classification of Education” (ISCED, 2011 version; [27]). It distinguishes between low (ISCED 0–2; respondents without completed vocational qualification and without A-levels (Abitur)), middle (ISCED 3–4; respondents with vocational qualifications or respondents with A-levels (Abitur) but no vocational qualifications or degrees) and high (ISCED 5–6; respondents with university degrees or master craftsmen) education. The place of residence was determined by matching the obtained register addresses against a database of nursing institutions classifying participants as living in a private home in the community or an institutionalized setting [27]. Participants were asked about their self-rated health status with the possibility to answer with “very bad”, “rather bad”, “rather good” and “very good”. Number of chronic diseases was assessed with 19 different questions about various diseases building a sum score for every participant.

### Statistical analysis

Descriptive statistics of the sample (unweighted) are provided in total and stratified by sex, age groups, marital status, place of residence, education, self-rated health status and number of chronic diseases. Proportions are given in percentages % (n) for the categorical and dichotomous variables, while the mean (standard deviation) is given for the continuous variables.

The prevalence of different levels of pain is determined by sex, age groups, marital status, place of residence and education, taking weighing factors into account [27]. Proportions per group are displayed in percentages % (95% confidence interval). A *p* value retrieved from a chi-square test indicates whether a significant relationship between pain and the above-listed variables exist.

In addition, a multinomial logistic regression analysis was conducted to test for significant relationships between sex, age groups, education, marital status, place of residence, self-rated health status as well as number of chronic diseases and the outcome of the categorical variable pain.

The statistical significance level was set to alpha = 0.05. Stata 17.0 software was used for all statistical analyses.

## Results

In the sample of 1863 participants, 50.2% were female, 40.6% were married, 91.9% lived in a private home and 52.2% had a medium education. In sum, 39.1% of the participants belonged to the age group 80–84 years, 33.6% of the participants were between 85 and 89 years old and 27.4% of the participants were ≥ 90 years (see Table 1). A “rather good” self-rated health status was reported by 50.8% of the participants. The mean score of number of chronic diseases was 3.5 (SD = 2.35) on a scale from 0 to 19.

**Table 1:** Descriptive statistics of the sample

	Total	Male	Female
	*N* = 1863	*N* = 927	*N* = 936
Age group			
80–84 years	39.08%	41.42%	36.75%
85–89 years	33.55%	32.25%	34.83%
≥ 90 years	27.38%	26.32%	28.42%
Marital status			
Married	40.55%	60.19%	21.07%
Married but separated, divorced, widowed, single	59.45%	39.80%	78.93%
Place of residence			
Private home	91.89%	95.47%	88.35%
Institution	8.11%	4.53%	11.65%
Education (ISCED 2011)			
Low	26.58%	10.43%	42.40%
Middle	52.16%	55.78%	48.61%
High	21.27%	33.79%	8.99%
Self-rated health status			
Very bad	7.22%	6.94%	7.50%
Rather bad	32.29%	32.54%	32.05%
Rather good	50.78%	51.52%	50.05%
Very good	9.70%	9.00%	10.40%
Number of chronic diseases (0–19)			
Mean (SD)	3.49 (2.35)	3.38 (2.24)	3.61 (2.44)

Not weighted. Marital status: 1 missing. Education: 134 missings. Self-rated health status: 8 missings. Number of chronic diseases: 20 missings

### Prevalence of pain

Regarding pain, data were available from 1851 participants. The prevalence of having no pain, moderate pain or severe pain was 28.5%, 45.1% and 26.4% respectively. For men, the prevalence of no or moderate pain was 30.9% and 48.2%, respectively, while the prevalence of women having no pain or moderate pain was 27.1% and 43.3%, respectively. The prevalence of severe pain in women was 29.6% whereas it was 20.9% for men. Further details are provided in Table 2.

**Table 2:** Prevalence rates: pain among different groups

	No pain	Moderate pain	Severe pain	*p* value
Total	28.50% (26.17–30.69)	45.06% (42.41–47.73)	26.44% (24.10–28.91)	
Sex				< 0.01
Male	30.93% (27.54–34.55)	48.22% (44.43–52.02)	20.85% (17.98–24.05)	
Female	27.12% (24.05–30.42)	43.25% (39.72–46.86)	29.63% (26.41–33.07)	
Age group				0.06
80–84 years	25.78% (22.49–29.38)	47.67% (43.69–51.68)	26.54% (23.10–30.29)	
85–89 years	32.59% (28.54–36.92)	42.09% (37.85–46.44)	25.32% (21.61–29.43)	
≥ 90 years	29.99% (25.38–35.04)	41.65% (36.60–46.87)	28.37% (23.76–33.47)	
Education				< 0.01
Low	27.89% (23.41–32.85)	40.55% (35.57–45.73)	31.56% (26.94–36.58)	
Middle	27.80% (24.60–31.24)	44.60% (40.87–48.39)	27.60% (24.22–31.26)	
High	32.23% (26.90–38.07)	53.66% (47.64–59.58)	14.10% (10.60–18.53)	
Marital status				0.29
Married	28.27% (24.76–32.06)	47.44% (43.36–51.56)	24.29% (20.89–28.04)	
Others	28.68% (25.64–31.94)	43.68% (40.24–47.18)	27.64% (24.56–30.94)	
Place of residence				0.09
Private	27.62% (25.25–30.13)	46.23% (43.48–49.02)	26.14% (23.74–28.69)	
Institution	35.48% (27.02–44.97)	35.73% (27.43–44.99)	28.78% (20.86–38.27)	

Weighing factor included; 95% CI in parentheses

The *p* value of the chi-square test indicates that there is a significant relationship between pain and sex as well as pain and education. The chi-square test did not find significant results for the relationship between pain and age group, marital status or place of residence.

### Multinomial logistic regression analysis

The multinomial logistic regression analysis included sex, age groups, education, marital status, place of residence, self-rated health status and number of chronic diseases as independent variables, while pain was used as the outcome variable.

Results in Table 3 show relative risk ratios (RRR) for different variables given the other variables in the model are held constant. The reference category for the outcome pain is no pain compared to either moderate pain or severe pain.

**Table 3:** Multinomial logistic regression model reporting relative risk ratio (RRR)

	Moderate pain		Severe pain	
Variables	RRR (SE)	95% CI	RRR (SE)	95% CI
Sex (reference: male)				
Female	1.11 (0.16)	0.85–1.46	1.54* (0.27)	**1.09**–**2.16**
Age (reference: 80–84 years)				
85–89 years	0.72* (0.10)	**0.55**–**0.95**	0.66* (0.12)	**0.46**–**0.95**
≥ 90 years	0.72* (0.11)	**0.53**–**0.97**	0.80 (0.16)	0.55–1.18
Education (reference: low)				
Middle	1.02 (0.16)	0.75–1.39	0.89 (0.16)	0.62–1.28
High	1.05 (0.20)	0.72–1.52	0.57* (0.14)	**0.35**–**0.92**
Marital status (reference: others)				
Married	0.90 (0.12)	0.69–1.18	0.99 (0.17)	0.71–1.39
Place of residence (reference: private)				
Institution	0.79 (0.19)	0.50–1.25	1.16 (0.32)	0.67–2.00
Self-rated health status (reference: very bad)				
Rather bad	2.03* (0.69)	**1.05**–**3.95**	0.67 (0.21)	0.36–1.24
Rather good	1.15 (0.37)	0.61–2.16	0.13** (0.04)	**0.07**–**0.23**
Very good	0.43* (0.15)	**0.21**–**0.87**	0.02** (0.01)	**0.01**–**0.06**
Number of chronic diseases	1.26** (0.04)	**1.18**–**1.34**	1.41** (0.05)	**1.31**–**1.52**

Reference: no pain

Observations included: 1750; standard errors in parentheses

**p < 0.01, *p < 0.05

For the model *comparing moderate pain to no pain*, significant results were obtained for the age categories 85–89 years and ≥ 90 years, self-rated health status and number of chronic diseases. The likelihood of moderate pain (reference: no pain) was associated with lower risks for the age groups 85–89 years (RRR 0.72, 95% CI 0.55–0.95) and ≥ 90 years (RRR 0.72, 95% CI 0.53–0.97), compared to the age group 80–84 years. A “rather bad” self-rated health status was associated with a higher risk (RRR 2.03, 95% CI 1.05–3.95) of moderate pain, whereas a “very good” self-rated health status was associated with a lower risk (RRR 0.43, 95% CI 0.21–0.87) of moderate pain. Furthermore, a higher number of chronic diseases was associated with a higher likelihood of moderate pain (reference: no pain; RRR 1.26, 95% CI 1.19–1.35). No significant associations were found for the variables sex, education, marital status, and place of residence.

For the model *comparing severe pain to no pain*, significant results were obtained for sex, age group 85–89 years, high education, self-rated health status and number of chronic diseases. The likelihood of severe pain (reference: no pain) was associated with lower risks for the age group 85–89 years compared to 80–84 years (RRR 0.67, 95% CI 0.47–0.96), high education compared to low education (RRR 0.55, 95% CI 0.34–0.90), a “rather good” (RRR 0.13, 95% CI 0.07–0.23) and “very good” (RRR 0.02, 95% CI 0.01–0.06) self-rated health status. Being female (RRR 1.53, 95% CI 1.09–2.16) and a higher number of chronic diseases (RRR 1.42, 95% CI 1.32–1.53) were associated with a higher likelihood of severe pain compared to no pain. No significant associations were found for the age group of ≥ 90 years old, middle education, marital status and place of residence.

## Discussion

### Main findings

Based on a large representative sample, this study aimed to investigate the prevalence and correlates of pain among the oldest old in Germany using representative data of the NRW80+ study. It was found that almost half of the participants experienced moderate pain and less than a third each reported no or severe pain, respectively. The likelihood of having moderate or severe pain was reduced among those who were older and presented with a better self-rated health but increased with a growing number of comorbidities. Severe pain was less likely among men and those with a higher education.

#### Prevalence

The prevalence of pain found in other studies differs partly from our findings. On one hand, Satghare et al. [29] found a prevalence of pain of 25.9% in Singaporeans participants aged 85 years or older which is much lower than in the present study. A European study found that 45.1% of those aged 75 years and older experienced pain [19]. On the other hand, Lehti et al. [5] showed a similar prevalence of pain of 61% in those aged 75 years and older in a Finnish sample. Differences in prevalence might occur because various scales were used to assess pain. Further, cultural aspects, socio-economic differences and access to medical resources might play a role since those studies were conducted across a variety of countries.

#### Age

Our results showed that older age decreased the likelihood of moderate and severe pain. However, previous research concluded that there is inconsistency in the effect of older age on less pain experience [30]. Pautex et al. [31] wrote that pain might be underreported in older age (referring to being older than 65 years) because of fear of negative treatment effects or the belief that pain is a normal experience. Li et al. [4] concluded from their slightly younger sample that pain prevalence increased until the age of 79 and decreased afterward. Similar results were achieved by Rapo-Pylkkö et al. [8] where the intensity of pain decreased with age. Possible additional explanations could be a lower pain perception caused by sensory dysfunction [4] or a higher survival rate of healthier older adults [8].

#### Health status

We showed in the present study that a better self-rated health status decreased the likelihood of moderate as well as severe pain. These results have been demonstrated in previous studies showing that worse self-rated health status is associated with a higher prevalence of pain [8] and the persistence of chronic pain [32–34]. This could be explained by the co-occurrence of several diseases at the same time not only causing pain but also negatively impacting the overall perceived health status.

#### Sex

In accordance with studies by Mallon et al. [20], Si et al. [6] and Patel et al. [15] covering ages above 85, above 60 and 65 years respectively, females had a higher risk of severe pain in our study. This clear consensus could be explained by a higher sensitivity to pain due to biological, psychological and social mechanisms. On the one hand, as women age, changes in female sex hormones affect their pain sensitivity, so that their pain threshold is lower than that of men [35]. On the other hand, stereotypical roles in society influence pain coping strategies and exposure in early life [36].

#### Education

According to our study, highly educated oldest old (compared to low education) have a decreased likelihood of severe pain. This was also shown in previous studies with younger participants, ranging from 50 to 75 years or older [6, 15, 19, 29]. It can be explained by a general additive impact of social disadvantage leading to more work-related injuries [37, 38] and low health literacy [37–39] leading to delayed doctor visits and therefore postponed treatments [40]. We assume that delayed treatments in turn are associated with higher pain levels. However, future research is required to test this assumption.

#### Number of chronic diseases

In terms of number of chronic diseases, previous studies including participants aged 50 years and older revealed that greater multimorbidity is positively associated with a higher prevalence and increase of pain [6, 15, 19], which is consistent with this current study. A possible explanation is that the oldest old suffer more and more from various diseases at the same time [41, 42] leading to pain at multiple sites [13, 43] which then increases the overall pain.

It should be emphasized that most of the existing studies mainly refer to samples of individuals aged 50, 60 or 65 years or older and did not differentiate between age groups. Therefore, our current study adds to our current knowledge by exclusively focusing on individuals aged 80 years and older.

### Strengths and limitations

The present study has several strengths and some limitations. First, our study extends previous scarce research about the prevalence of pain and its correlations among the oldest old by exclusively examining individuals aged 80 years and older, also including individuals living in nursing homes. Furthermore, the NRW80+ generally has a high methodological quality enabling the oldest old population of North Rhine-Westphalia, Germany, to be represented. Moreover, weights were applied. Additionally, the way of measurement of the outcome variable pain was comparable to results from other existing studies. However, future research should make use of even more nuanced tools to measure pain.

Limitations include the cross-sectional study design of the NRW80+ study which makes it difficult to clarify the directionality and make assumptions about the causality of the associations. Future research using a longitudinal design is desirable. Moreover, there may be a slight selection bias in the NRW80+ study since the response rate was rather low. For example, it may be the case that non-responders have a higher likelihood of being less healthy (including higher pain levels) compared to responders.

## Conclusion

Study findings based on a large representative sample showed that almost half of the participants experienced moderate pain and less than a third each reported no or severe pain, respectively. Being 85 years or older (compared to being between 80 and 84 years old) and a better self-rated health status decreased the likelihood of moderate pain. Compared to men, females were found to have a higher risk of severe pain, but not moderate pain. Being 85–89 years old, higher education and a better self-rated health status decreased the likelihood of severe pain. However, both moderate and severe pain, were more likely to occur with a higher number of chronic diseases.

To gain a deeper understanding of aging, future studies could utilize a longitudinal design to detect changes within individuals as they continue to age. This approach would also help to establish causal relationships between various factors. Additionally, it should be continued to include people living in institutions to enable comparisons between them and community-dwelling oldest old individuals.

It could be assumed that a higher prevalence of pain indicates a lower quality of life which could affect various aspects of the oldest old daily life like independence and autonomy [44]. Furthermore, pain might be kept to a minimum or even prevented if a healthier lifestyle would be promoted by educating people which also lowers the prevalence of comorbidities [18, 39, 44]. Pain management could be improved by breaking up stereotypes at an earlier age [36]. Addressing pain not only has the potential to enhance overall well-being of the oldest old but could also lead to positive effects on mental health and social engagement.

## Data Availability

The datasets generated and/or analysed during the current study are publicly available at https://search.gesis.org/research_data/ZA7558.
